# Liberal versus restrictive transfusion strategies in acute myocardial infarction: a systematic review and comparative frequentist and Bayesian meta-analysis of randomized controlled trials

**DOI:** 10.1186/s13613-024-01376-1

**Published:** 2024-09-28

**Authors:** Rayan Braïk, Safa Jebali, Pierre-Louis Blot, Julia Egbeola, Arthur James, Jean-Michel Constantin

**Affiliations:** 1 Service de Réanimation Chirurgicale Polyvalente, Sorbonne University, GRC 29, AP-HP, DMU DREAM, and Department of Anaesthesiology and Critical Care, Pitié-Salpêtrière Hospital, 47-83 Bd de L’Hôpital, 75013 Paris, France; 2 Paris Saclay University, AP-HP, Bicêtre Hospital, Le Kremlin-Bicêtre, France

**Keywords:** Myocardial infarction, Blood transfusion, Randomized controlled trials, Anemia, Metanalyse

## Abstract

**Background:**

The transfusion strategy in the acute phase of myocardial infarction (AMI) remains a debated topic with non-standardized guidelines. This study aimed to evaluate the impact of liberal versus restrictive transfusion strategies on mortality during AMI.

**Methods:**

A systematic search was conducted across MEDLINE, EMBASE, and the COCHRANE library databases, focusing on randomized controlled trials (RCTs). The primary endpoint was the latest measured mortality within 90 days following myocardial infarction (MI). Secondary endpoints included recurrence of MI, cardiovascular mortality, stroke occurrence, unplanned revascularization, and a composite endpoint of death or recurrent MI. Mixed and random-effects models were employed to estimate relative risks. Sensitivity analyses were conducted using two approaches: one incorporating only studies assessed as low risk of bias according to the Rob2 tool, and another employing a Bayesian analysis.

**Results:**

Four RCTs including a total of 4324 participants were analyzed. Neither the fixed-effect nor random-effects models demonstrated a significant reduction in mortality, with risk ratios (RR) of 1.16 (95% CI 0.95–1.40) for the fixed-effect model and 1.13 (95% CI 0.67–1.91) for the random-effects model (GRADE: low certainty of evidence). Sensitivity analyses, including the exclusion of two high-risk-of-bias studies and a Bayesian analysis, were consistent with the primary analysis. For the composite outcome death or MI both fixed-effect and random-effects models showed a statistically significant RR of 1.18 (95% CI 1.01–1.37) with negligible heterogeneity (I^2^ = 0%, p = 0.46), indicating results unfavorable to restrictive transfusion (GRADE: very low certainty of evidence). However, this result was primarily driven by a single study. For cardiac mortality, the fixed-effects model indicated a significant RR of 1.42 (95% CI 1.07–1.88), whereas the random-effects model showed non-significant RR of 1.05 (95% CI 0.36–3.80). Analyses of other secondary endpoints did not show statistically significant results.

**Conclusions:**

Our analysis did not demonstrate a significant benefit in early mortality with a liberal transfusion strategy compared to a restrictive strategy for AMI, low certainty of evidence. Liberal transfusion may reduce the risk of the composite outcome death or MI, with very low certainty of evidence. These findings should be interpreted with caution in critically ill patients.

**Supplementary Information:**

The online version contains supplementary material available at 10.1186/s13613-024-01376-1.

## Introduction

The management of acute myocardial infarction (AMI) has evolved significantly with the adoption of thrombolytics, antiplatelet therapies and percutaneous coronary interventions, significantly improving patient outcomes [1]. However, this advancement has also led to an increased incidence of bleeding complications, frequently resulting in anemia and the subsequent requirement for blood transfusions [2, 3]. Anemia is frequently observed in AMI patients and worsens myocardial injury by intensifying the mismatch between oxygen supply and demand [3]. This underscores the role of red blood cell transfusion in restoring hemodynamic stability and enhancing oxygen delivery to ischemic myocardial tissues [4].

Anemia is commonly encountered in the intensive care unit (ICU), particularly among patients with extended hospitalizations [5]. Concurrently, elevated troponin levels are frequently observed, often indicative of type 2 myocardial infarction (MI). Research indicates that troponin elevation rates in ICUs frequently exceed 40%, underscoring the prevalent cardiovascular stress experienced by these patients [6]. Additionally, individuals admitted with AMI typically require relocation to specialized monitored units, ensuring focused care and continuous monitoring [1].

The decision-making process regarding transfusions in AMI is complex. While transfusions are aimed at improving oxygenation, they are associated with a range of potential adverse effects that can have a significant impact on patient outcome. Notably, there is an increased risk of thrombosis due to the hypercoagulable state often seen in AMI patients. Additionally, respiratory complications such as transfusion-related acute lung injury (TRALI) and pulmonary edema potentially outweighing the beneficial effects of transfusion [7, 8].

Transfusion thresholds guiding the management of the early phase of AMI remains debated [9, 10]. The latest 2020 ESICM guidelines advocate for a liberal transfusion strategy during the acute phase of AMI. However, these guidelines have not yet integrated findings from two significant multicenter studies published subsequently in 2021 and 2023 [11, 12]. Our work aimed to update the scientific knowledge base on this topic. Due to the limited number of publications included in a previous meta-analysis, a Bayesian analysis was employed to provide a more comprehensive understanding of the data (see Table 1).

**Table 1:** Comparative overview of study characteristics in transfusion strategy meta-analysis

Study	Carson et al. 2023	Ducrocq et al. 2021	Carson et al. 2013	Cooper et al. 2011
Participants	3504	666	110	45
Inclusion Criteria	Myocardial infarction with anemia < 10 g/dl	Myocardial infarction, Hb between 7 and 10 g/dL	Myocardial infarction, unstable angina, stable coronary disease, Hb < 10 g/dL	Myocardial infarction, hematocrit ≤ 30%
Exclusion Criteria	Uncontrolled bleeding, palliative treatment, scheduled cardiac surgery, decline of blood transfusion	Shock myocardial infarction post-PCI or CABG, blood transfusion in last 30 days, malignant hematologic disease	Active bleeding, hemodynamic instability, health issues interfering with symptom reporting or treatment adherence	Non-coronary cause, active bleeding (≥ 5% hematocrit drop in 12 h), unwillingness/inability to receive RBC transfusion, recent RBC transfusion (within 7 days), severe transfusion reaction history, imminent death, limited/comfort care decision, age < 21
Baseline Characteristics (Restrictive Group)	Age: 72.2 years Female: 44.3% Ethnicity: 70.3% white NSTEMI: 81.8% Type 1: 41.7% Type 2: 55.3% Revascularization: 29.1% Heart failure: 21.6% Dialysis: 11.8% Mechanical ventilation: 13.7%	Age: 78 years Female: 41.2% Ethnicity: 88.7% white NSTEMI: 68.4% Revascularization: 58.8% Dialysis: 8% Mechanical ventilation: NA	Age: 74.3 years Female: 50.9% Ethnicity: 74.6% white NSTEMI: 47.3% Revascularization: 47.3% Heart failure: 23.6% Dialysis: NA Mechanical ventilation: NA	Age: 70.3 years Female:46% Ethnicity: 61% white NSTEMI: 54% Revascularization: 54% Heart failure: NA Dialysis: NA Mechanical ventilation: 13%
Baseline Characteristics (Liberal Group)	Age: 72.1 years Female: 46.7% Ethnicity: 70.9% white NSTEMI: 80.8% Type 1: 41.6% Type 2: 56.3%, Revascularization: 28.1% Heart failure: 23%	Age: 76 years Female: 43.2% Ethnicity: 82.6% white NSTEMI: 71.3%, Revascularization: 59.3%	Age: 67.3 years Female: 49.1% Ethnicity: 70.9% white NSTEMI: 38.2% Revascularization: 63.6%Heart failure: 21.8%	Age: 76.4 years Female:52% Ethnicity: 76% white NSTEMI: 67% Revascularization: 57% Heart failure: NA Dialysis: NA Mechanical ventilation: 24%
Hemoglobin levels	Baseline: 8.6 g/dl for restrictive 8.6 g/dl liberal Days 3: 8.9 g/dl for restrictive 10.5 g/dl liberal Discharge: NA	Baseline: 9 g/dl for restrictive 9.1 g/dl liberal Days 3: NA Discharge: 9.7 g/dl for restrictive 11,1 g/dl for liberal	Baseline: 9.03 g/dl for restrictive 10.3 g/dl liberal Days 3: 9.12 g/dl for restrictive 10.64 g/dl liberal Discharge: NA	Baseline: 27.5% for restrictive 26.9% liberal Days 3: ~ 27–28% for restrictive ~ 32–33% for liberal Discharge: ~ 27–28% for restrictive ~ 28–30% for liberal
ICU at randomization	47.9%	NA	NA	NA
Countries and Sites	US, Canada, France, Brazil, New Zealand, Australia (144 sites)	France, Spain (35 sites)	US (8 sites)	US (3 sites)
Group Details (Restrictive)	Blood transfusion permitted under 8 g/dl	No transfusion unless Hb ≤ 8 g/dL, target post-transfusion Hb 8–10 g/dL	One unit of RBCs post-randomization, transfusion if Hb < 10 g/dL	Transfusion was initiated when hematocrit fell below 24%, aiming to maintain levels between 24 and 27%
Group Details (Liberal)	One unit of RBCs post-randomization, maintain Hb ≥ 10 g/dL	Transfusion for Hb ≤ 10 g/dL, target post-transfusion Hb ≥ 11 g/dL	Transfusion allowed for anemia-related symptoms, no mandatory lower threshold	Transfusion was initiated when hematocrit fell below 30%, aiming to maintain levels between 30 and 33%
Overall risk of bias (Rob-2)	Low risk of bias	Low risk of bias	High risk of bias	High risk of bias
Study Dates	April 2017–April 2023	March 2016–September 2019	March 2010–May 2012	May 2003–October 2009

This table presents a comprehensive overview of the key characteristics of the three studies included in our meta-analysis: Carson et al. 2023, Ducrocq et al. 2021, Carson et al. 2013 and Cooper et al. 2011

MI: myocardial infarction; Hb: hemoglobin; PCI: percutaneous coronary intervention; CABG: coronary artery bypass grafting; NSTEMI: non-ST-elevation myocardial infarction; RBCs: red blood cells; Rob-2: revised Cochrane risk-of-bias tool for randomized trials.

## Materiels and methods

### Study design and registration

This study was registered in PROSPERO (CRD42024483286) and conducted in accordance with the PRISMA guidelines (Preferred Reporting Items Systematic Reviews and Meta-Analysis) [13]. The PRISMA checklist is provided in the supplementary materials (supplementary Document 1). We adopted the PICO framework to define our research question and inclusion criteria: Patients (P) were adults hospitalized with AMI; the Intervention (I) was a liberal threshold for transfusion; the Comparator (C) was a restrictive threshold for transfusion; and the Outcome (O) of interest was mortality.

### Definitions

This study included adult patients hospitalized for the management of AMI, defined according to standardized criteria. We incorporated all types of acute AMI, including Type 1, Type 2, Type 3, Type 4, and Type 5, as well as both STEMI (ST-segment elevation myocardial infarction) and non-STEMI (non-ST-segment elevation myocardial infarction) cases in our meta-analysis [4]. Studies focusing on AMI in the perioperative context of cardiac surgery were excluded from our analysis. This exclusion was implemented to ensure a homogeneous patient profile.

In this study, the restrictive transfusion threshold was defined below hemoglobin levels of 7 to 8 g/dL. The liberal transfusion threshold was set at hemoglobin levels below 9 to 10 g/dL. The primary outcome was defined as the latest measured mortality within 90 days or during hospitalization. However, none of the studies reported mortality at 90 days; the longest follow-up for mortality was 30 days. Secondary outcomes included the incidence of a new MI, the need for emergency revascularization, stroke occurrence, and a composite endpoint of early death or MI.

### Data source and search strategy

Our analysis included exclusively randomized controlled trials (RCT) published in English or French. We excluded letters, conference abstracts, and research letters. The specific search equation is provided in the supplementary materials (S1). MEDLINE, EMBASE and the COCHRANE library databases were used for our search. Two independent blind reviewers (RB and SJ) conducted an initial screening based on the titles and abstracts. In case of disagreement, a third blind reviewer (PLB) provided a decisive opinion. The full text screening was then performed following the same blinded process, involving a thorough review of the full manuscripts. To limit bias inherent to changes in clinical practices, the bibliographic search was restricted to studies published after 2000. The process of study selection was facilitated using Rayyan, a web-based software designed to streamline the systematic review process [14].

### Data extraction and quality assessment

The biases of each study and each outcome were evaluated using the Risk of Bias 2 (RoB 2) tool [15]. RoB 2assesses biases across five domains: bias arising from the randomization process, bias due to deviations from intended interventions, bias due to missing outcome data, bias in measurement of the outcome, and bias in selection of the reported result. Each identified bias is then categorized as ‘low risk’, ‘high risk’, or ‘some concerns’. One reviewer performed the data extraction on the manuscripts and supplementary materials, while the other conducted a thorough check for accuracy and completeness (RB and PLB).

### Statistical analysis

This meta-analysis was conducted following the Cochrane RevMan5 settings using the R package ‘meta’ [16]. We applied both fixed and random-effects models to analyze the data, presenting the results as relative risks. Heterogeneity among the studies was assessed using the I^2^ statistic. I^2^ values between 0 and 30% indicate low heterogeneity, potentially negligible; I^2^ values between 30 and 50% represent moderate heterogeneity; I^2^ between 50 and 75% defines substantial heterogeneity; and I^2^ between 75 and 100% suggests considerable heterogeneity. Additionally, the Cochrane Q test was used to evaluate heterogeneity, considering statistical significance at P < 0.05. The visual assessment of publication bias was conducted using a Funnel plot. Due to the limited number of studies included, statistical tests for publication bias were not conducted, as the sample size was insufficient for reliable conclusions in this context. The details of the statistical methodology are provided in the supplementary materials. To strengthen the robustness of our findings, we performed a sensitivity analysis, focusing exclusively on studies categorized as having a low risk of bias (Rob-2). Given the relatively small sample size and the objective to minimize assumptions, a Bayesian probabilistic approach was incorporated into the sensitivity analysis. This involved using Markov chain Monte Carlo methods, selecting a binomial likelihood function, and applying a random-effects model with non-centered parametrization [17]. The selection of prior distributions for μ (mean), θ (standard deviation), and τ (heterogeneity)—μ centered at 0 with a standard deviation of 10, and τ set at 0.5—was intended to capture a broad range of potential effect sizes and a moderate heterogeneity expectation, consistent with minimal baseline assumptions. Detailed parameters of this approach are provided in the supplementary materials. The quality of evidence was evaluated, using the GRADE approach (The Grading of Recommendations Assessment, Development, and Evaluation) [18]. All statistical analyses were conducted using R software, version 4.3.2. All extracted data and the corresponding R code for analyses are available in the supplementary materials for transparency and reproducibility.

## Results

### Study selection

**Fig. 1:**
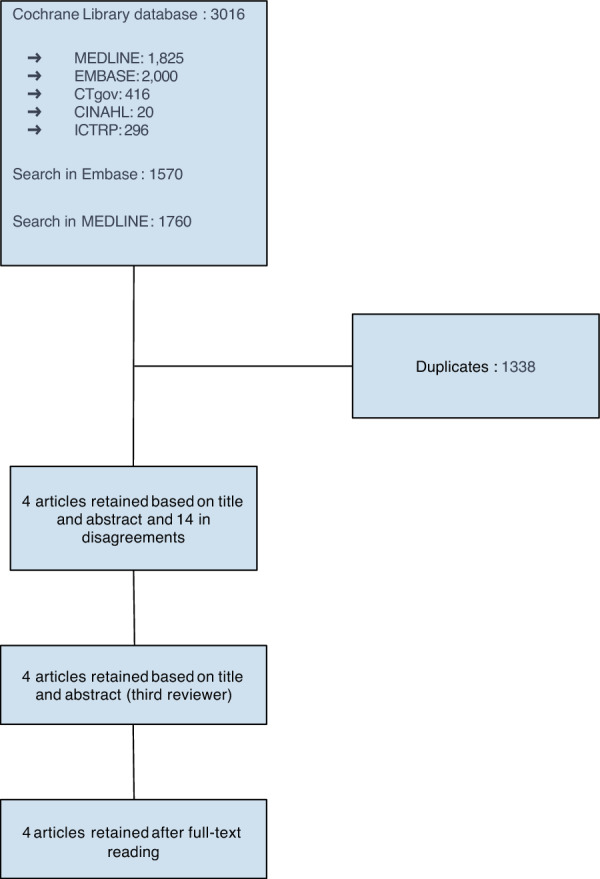
Flowchart of study selection process for the meta-analysis. This flowchart outlines the study selection process for our meta-analysis. From an initial pool of 6346 articles, duplicates were removed, followed by title and abstract screening. Full-text reviews were then conducted to assess eligibility, resulting in the inclusion of 4 studies in the meta-analysis

A total of 6346 articles were identified through bibliographic search. Among these, 1760 were found on MEDLINE, 1570 on EMBASE, and 3,016 via the Cochrane Library (including overlaps of 1825 articles on MEDLINE, 2000 on EMBASE, 416 on CTgov, 2 on CINAHL, and 296 on ICTRP). A total of 1338 duplicates were removed. Following an initial screening based on title and abstract, 4 articles were selected (with 14 excluded after consultation with the third reviewer). Upon full-text review, 4 articles were included [11, 12, 19, 20]. Details of study selection are mentioned in Fig. 1**.**

### Primary outcomes

**Fig. 2:**
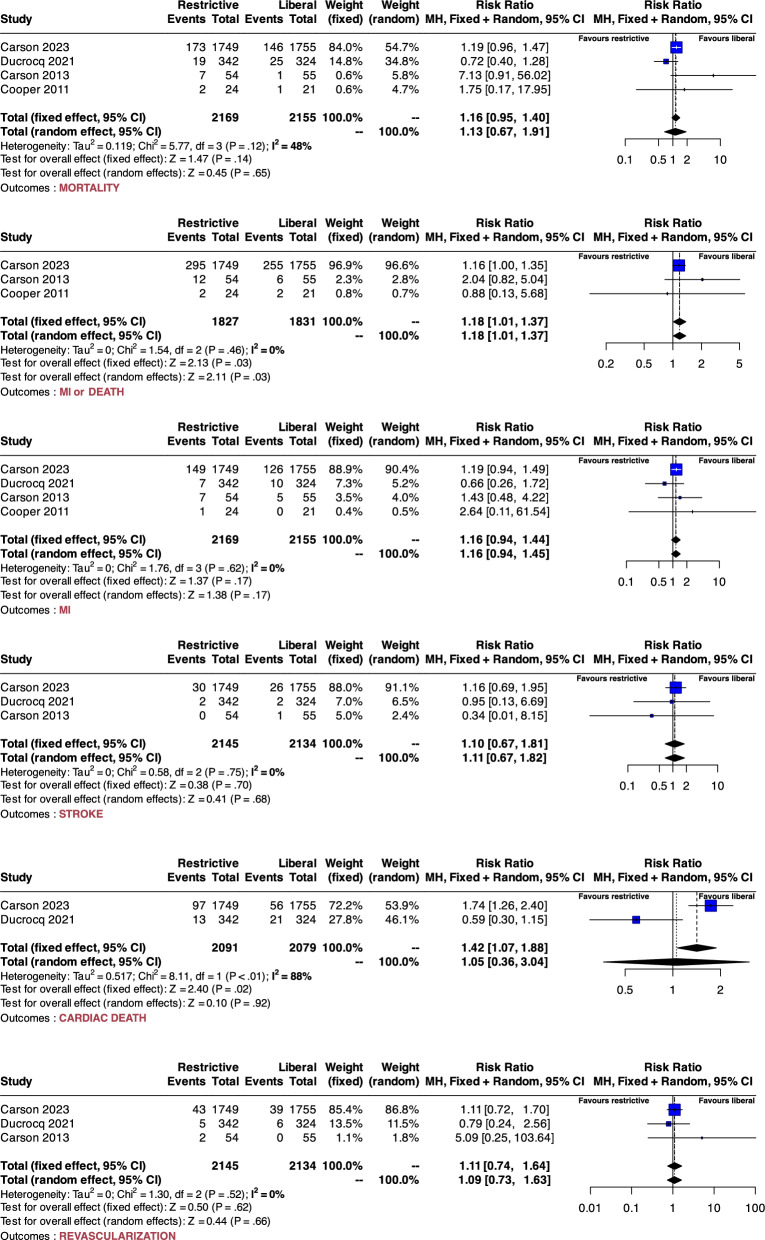
Forest plot from meta-analysis. The plot illustrates the results of our meta-analysis, conforming to Cochrane RevMan5 settings, including both fixed and random-effects models showing relative risks for each included study. Heterogeneity is assessed using the I^2^ statistic, where an I^2^ value above 50% indicates significant heterogeneity. The plot also includes results of the Cochrane Q test for heterogeneity, with a significance threshold of P < 0.05. Mortality in this context is defined as early mortality, occurring either within 90 days or during hospitalization. The primary endpoint was the latest measured mortality within 90 days following myocardial infarction. Secondary outcomes were defined as the incidence of a new myocardial infarction (MI), the need for emergency revascularization, the occurrence of stroke, and a composite endpoint of early death or MI. MI: myocardial infarction; RR: risk ratio

The analysis of the mortality outcome included four studies, involving a total of 4324 patients. The results showed no significant difference between the liberal and restrictive protocols, with an estimated risk ratio (RR) of 1.16 (95% CI 0.95 to 1.40) for the fixed-effect model and 1.13 (95% CI 0.67 to 1.91) for the random-effects model with moderate heterogeneity (I^2^: 48%, p = 0.12) (see Fig. 2).

### Secondary outcomes

For the composite outcome death or MI, the fixed-effects and random effects model showed a statistically significant RR of 1.18 (95% CI 1.01 to 1.37) in favor of the liberal strategy, with negligible heterogeneity (I^2^: 0%, p = 0.46). For recurrence of MI, both fixed and random-effects models yielded non-significant RRs of 1.16 (95% CI 0.94 to 1.44) and 1.16 (95% CI 0.94 to 1.45) with negligible heterogeneity (I2: 0%, p = 0.62).

For strokes, both fixed and random-effects models showed non-significant RRs of 1.10 (95% CI 0.67 to 1.81) and 1.11 (95% CI 0.67 to 1.82), respectively, with negligible heterogeneity (I2: 0%, p = 0.75). For cardiac death, the fixed-effects model showed a statistically significant RR of 1.42 (95% CI 1.07 to 1.88) in favor of the liberal strategy. However, this finding exhibited inconsistency when analyzed using the random-effects model, which reported an RR of 1.05 (95% CI 0.36 to 3.04) with considerable heterogeneity (I2: 88%, p < 0.01). For revascularization, both fixed and random-effects models showed non-significant RRs of 1.11 (95% CI 0.74 to 1.64) and 1.09 (95% IC: 0.73 to1.63) respectively, with negligible heterogeneity (I2: 0%, p = 0.52). Details are provided in Fig. 2.

### Sensitivity analysis

**Fig. 3:**
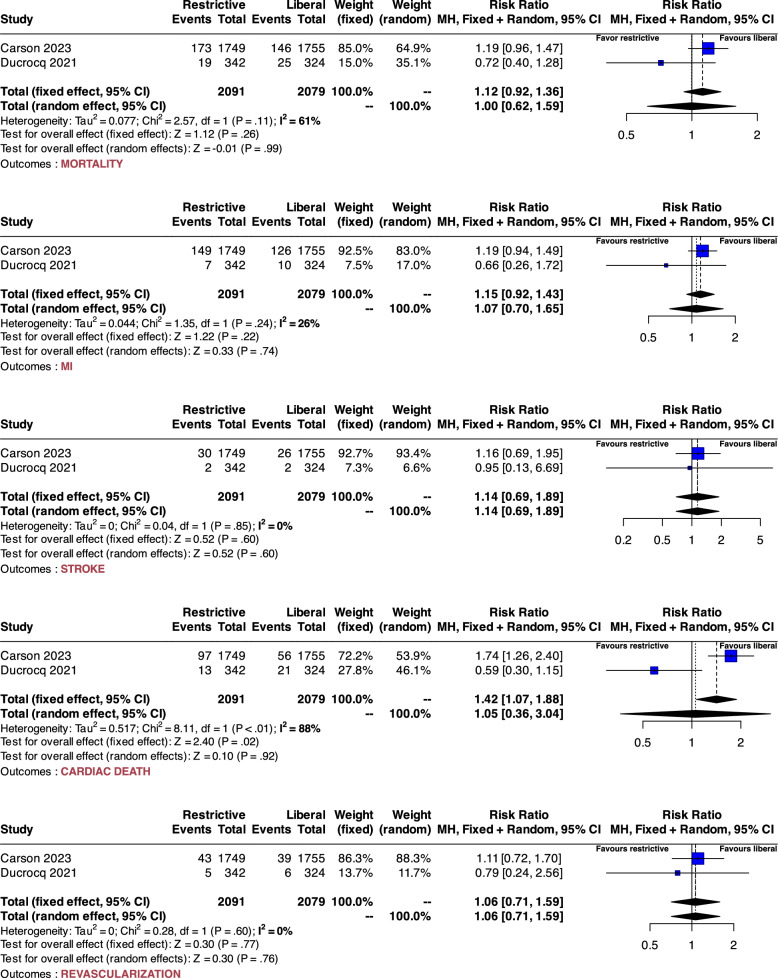
Sensitivity analysis forest plot. The plot illustrates the results of a sensitivity analysis, conforming to Cochrane RevMan5 settings, including both fixed and random-effects models showing relative risks for each included study. For this analysis, we included only those studies deemed to have a low risk of bias for each criterion according to the Rob-2 tool. Heterogeneity is assessed using the I^2^ statistic, where an I^2^ value above 50% indicates significant heterogeneity. The plot also includes results of the Cochrane Q test for heterogeneity, with a significance threshold of P < 0.05. Mortality in this context is defined as early mortality, occurring either within 90 days or during hospitalization. The primary endpoint was the latest measured mortality within 90 days following myocardial infarction. Secondary outcomes were defined as the incidence of a new myocardial infarction (MI), the need for emergency revascularization, the occurrence of stroke, and a composite endpoint of early death or MI. MI: myocardial infarction; RR: risk ratio

**Fig. 4:**
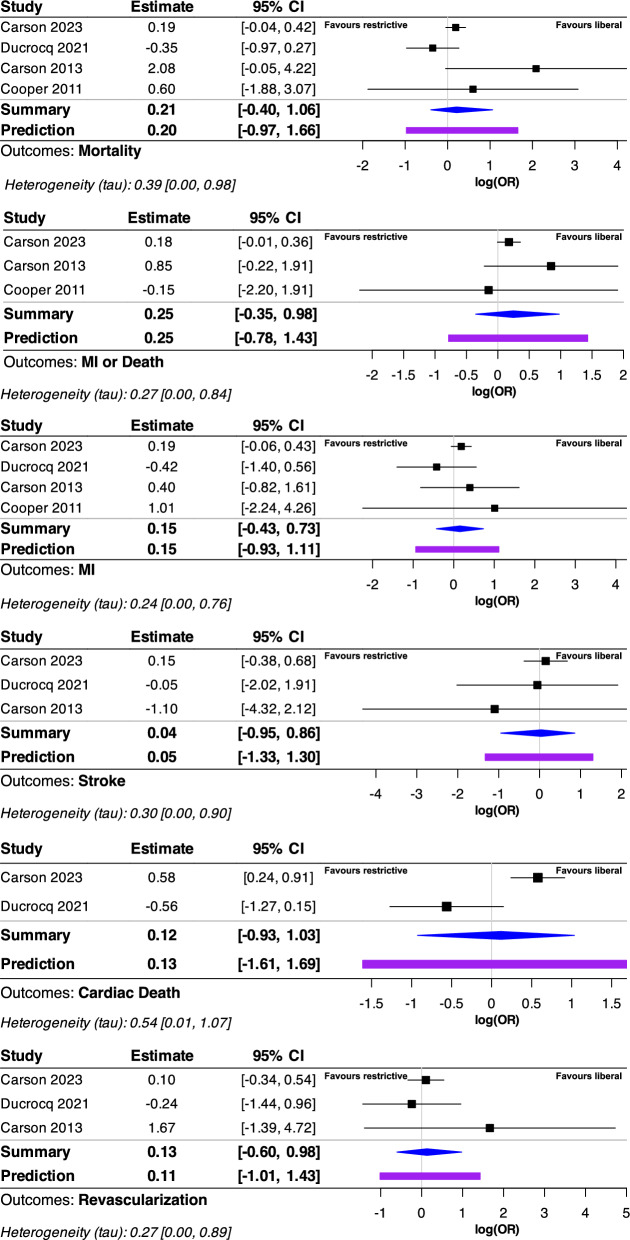
Bayesian sensitivity analysis forest plot. This figure shows the results of a Bayesian sensitivity analysis conducted using Markov chain Monte Carlo (MCMC) methods within a random-effects model and non-informative priors. The top section of the forest plot displays the log odds ratio estimates and their 95% confidence intervals for each study included. In the lower portion, the initial number (summary) quantifies our current understanding of the treatment effect size from the existing data. The number below it (predictions) provides estimates for what outcomes might be expected if new, similar studies were to be conducted. Both are presented with their 95% credible intervals and depicted as a posterior probability distribution of the log odds ratio (logOR)

A sensitivity analysis was performed after excluding two studies (Carson 2013 and Cooper 2011) due to their high risk of bias. For mortality, both fixed and random-effects models showed non-significant RRs of RR 1.12 (95% IC: 0.92 to 1.36) and 1.0 (95% IC: 0.62 to 1.59), respectively. Details are provided in Fig. 3. The Bayesian probabilistic sensitivity analysis revealed that the distribution of the log posterior probability of the estimated odds ratio (OR) ranges from -0.97 to 1.66. (see Fig. 4). For the composite outcome death or MI, the distribution of the log posterior probability of the estimated OR ranges from -0.78 to 1.43. For the other secondary outcomes, the sensitivity analysis findings are consistent with the primary analysis results. Details are provided in Figs. 3 and 4.

### Study quality assessments

The Rob-2 tool identified Carson’s 2013 [19] study as having a high risk of bias for several key outcomes: mortality, occurrence of secondary MI, the composite outcome of MI or death, cardiovascular-related deaths, stroke, and unplanned revascularization (see supplementary Fig. 1). This high-risk designation in Domain 2 (deviation for intended interventions) was primarily attributed to an inappropriate analysis used to estimate the intervention's effect, coupled with a potential for a significant impact on the study’s outcomes due to non-compliance, with 6.3% of patients not receiving their allocated interventions with low incidence of outcome. Furthermore, the randomization process raised concerns, primarily driven by significant baseline differences in age and severity of the included patients. Similarly, Cooper's 2013 study was also identified as having a high risk of bias for several key outcomes, such as mortality, MI or death and secondary MI. This high-risk designation in Domain 2 was attributed to inappropriate analysis methods and significant non-compliance, with 6.6% of patients not receiving their allocated interventions, potentially impacting outcomes. The randomization process raised concerns, primarily due to significant baseline differences in mechanical ventilation. Details on the risk of bias are included in supplementary Document 2.

### Certainty of the evidence

We employed the Grading of Recommendations Assessment, Development, and Evaluation (GRADE) system to assess the certainty of evidence [18]. We classified the certainty of evidence as 'low' for mortality and for the recurrence of MI. All other outcomes were deemed to have a 'very low certainty of evidence’. Comprehensive details of this GRADE assessment can be found in supplementary Table 1.

## Discussion

This meta-analysis of randomized controlled trials did not show a significant benefit in early mortality with a liberal transfusion strategy compared to a restrictive strategy for AMI, with low certainty of evidence. These results are consistent with sensitivity analyses, including only studies with a low risk of bias and Bayesian sensitivity analysis. Frequentist mixed and random models indicated that liberal transfusion might reduce the risk of the composite outcome of death or MI. However, sensitivity analysis with no risk of bias could not be conducted as there was only one low-risk study available. Additionally, the Bayesian analysis was inconsistent with the frequentist analysis.

In this context, we conclude that liberal transfusion may decrease the risk of early mortality or MI, with very low certainty of evidence (GRADE). For the other secondary outcomes, including recurrent MI, need for revascularization, stroke, and cardiac death, we concluded that liberal transfusion may or may not be beneficial, with very low certainty of evidence (GRADE).

One important point to highlight from our meta-analysis is the moderate to high heterogeneity for certain outcomes. Secondly, most of the findings are driven by two studies: REALITY and the MINT trial. Additionally, some outcomes, such as composite mortality and MI, are predominantly influenced by the MINT trial [11, 12]. REALITY focused on mortality or major cardiac events, while the MINT trial concentrated on death or MI, with resulting relative risks of 0.78 (95% CI 0.0–1.17) and 1.16 (95% CI 1–1.35), respectively. Despite similar protocols for transfusion thresholds and participant demographics, these differing outcomes highlight the complexity of applying transfusion strategies in acute MI. Both studies targeted hemoglobin levels of 8 to 10 g/dl in restrictive groups and over 10 g/dl in liberal groups, with comparable age, ethnicity, and proportions of NSTEMI among participants. However, patients in the REALITY trial had more revascularizations before randomization than those in the MINT trial. Additionally, 36% of patients in the MINT study received transfusions before randomization, whereas the REALITY study did not allow the inclusion of patients who had received transfusions in the last 30 days.

Recent discussions have raised concerns about the suitability of a 7% p-value threshold in the MINT trial, suggesting that the inclusion of just four additional participants might have led to statistically significant results [21]. Our frequentist analysis showed a significant increase in the risk of death or MI, in case of a restrictive strategy. However, it is important to highlight that these results are primarily driven by the MINT trial, which accounted for 96.6% of the total weight of the results. The other two studies included in this analysis (Carson 2013 and Cooper 2011) were considered to be at high risk of bias. Therefore, a sensitivity analysis could not be performed based on only one low-risk study. Additionally, the Bayesian inference with non-informative priors was inconsistent with the frequentist analysis. These results could be influenced by the nature of the non-informative priors we chose. Consequently, it is important to note that, according to the GRADE assessment, the level of evidence was rated as “very low certainty of evidence”.

Although we did not demonstrate a significant reduction in early mortality (GRADE: very low certainty of evidence), it is important to note that our primary outcome was measured mortality within 90 days or during hospitalization. However, none of the studies reported mortality at 90 days and the longest follow-up for mortality was 30 days. Although early mortality was high (8.6%), the relevance of 30-day mortality is questionable because one of the major issues after an AMI is the development of chronic heart failure. Furthermore, two recent ancillary analyses of the REALITY trial have been conducted. One revealed a significant increase in long-term mortality among heart failure patients in the liberal transfusion group [22]. The second showed that the major adverse cardiovascular event (MACE) criterion did not achieve clinical noninferiority at 1 year [23].

A second limitation, in the interpretation of our meta-analysis’ results is the lack of sequential hemoglobin measurements in the MINT and REALITY studies. Specifically, the MINT study documented baseline hemoglobin levels of 8.6 g/dL for both groups, with levels rising to 10.5 g/dL and 8.9 g/dL by Day 3. Conversely, the REALITY study reported initial hemoglobin levels of 9 and 9.1 g/dL, median minimum values of 8.3 and 8.8 g/dL for the restrictive and liberal groups respectively, and discharge levels of 9.7 and 11.1 g/dL. The relatively small differences between the restrictive and liberal groups in hemoglobin levels noted in the REALITY study, particularly with values approaching 9–10 g/dL, could explain the absence of significant effects observed in this study.

Another limitation of our study concerns the generalization of our results to patients in the intensive care setting. Specifically, the MINT study included approximately 48% of patients in ICUs at randomization. Additionally, 13.7% of these patients were on mechanical ventilation, 13% exhibited active bleeding, 23% suffered from congestive heart failure, and 11.8% required dialysis. Given the similar patient characteristics reported in the REALITY study, we anticipate a comparable proportion of patients admitted in the intensive care. Nevertheless, we advise caution in extending our findings to severely ill ICU patients, especially those in shock. However, we believe our results may still be relevant to less critically ill ICU profiles or patients in specialized monitoring units.

The 2020 ESCIM guidelines recommend a transfusion threshold of 9–10 g/dL for critically ill adults with acute coronary syndrome, supported by low certainty of evidence[24]. These guidelines are based in part on earlier studies such as Carson 2013 and the CRIT pilot, but do not include findings from more recent studies like the MINT and REALITY trials. Because of this, it is important to update the evidence to include recent data. Also, the lack of individualized patient parameters in the studies we reviewed shows the need for a more pathophysiological approach. Current transfusion practices, mainly based on hemoglobin levels or symptoms, may not fully address the complexity of myocardial injury. A better approach could involve adjusting transfusion strategies based on indicators like myocardial oxygen consumption and distress, such as cardiac biomarkers, myocardial perfusion imaging, rather than just hemoglobin levels and symptoms.

The results of our meta-analysis regarding mortality and our secondary judgement criteria are consistent with the two recently published meta-analyses on this topic by Sukhon et al*.*, and the updated meta-analysis by Mistry et al*.* [26, 27]. Unlike these recent studies, we employed a more restrictive approach by excluding studies with high and intermediate risk of bias. Bayesian analysis conducted in the context of a small number of included studies, aligns with the frequentist analysis and results published in recent meta-analyses.

## Conclusion

Our analysis did not demonstrate a reduction in early mortality with a liberal transfusion strategy compared to a restrictive strategy, with low certainty of evidence. Liberal transfusion may reduce the risk of the composite outcome of death or myocardial infarction, with very low certainty of evidence. These findings should be interpreted with caution in critically ill patients. Further individualized studies based on a pathophysiological approach may be useful to fully explore this question.

## Supplementary Information

Additional file 1.

Additional file 2.

Additional file 3.

Additional file 4.

Additional file 5.

Additional file 6.

Additional file 7.

Additional file 8. Figure S1. Funnel Plot Analysis for Heterogeneity and Publication Bias Assessment. This funnel plot was employed to visually assess heterogeneity and potential publication bias in our meta-analysis.

Additional file 9. Figure S2. Rob2 Risk-of-Bias Visualization. This graphic displays the risk-of-bias for each study and each outcome in our meta-analysis as per Rob2 criteria (revised Cochrane risk-of-bias tool for randomized trials). It categorizes bias risk levels across key domains for each study, allowing for a quick comparative assessment of their methodological quality.

## Data Availability

All extracted data and the corresponding R code for the analyses are available in the supplementary materials.

## Abbreviations

AMI: Acute myocardial infarction
GRADE: Grading of recommendations assessment, development, and evaluation
ICU: Intensive care unit
MI: Myocardial infarction
NSTEMI: Non-ST-segment elevation myocardial infarction
OR: Odds ratio
PROSPERO: International prospective register of systematic reviews
PRISMA: Preferred reporting items for systematic reviews and meta-analyses
PICO: Patient, intervention, comparator, outcome
RoB: Risk of bias
RR: Relative risk
RCTs: Randomized controlled trials
STEMI: ST-segment elevation myocardial infarction
