# Supplementary material for: Liberal versus restrictive transfusion strategies in acute myocardial infarction: a systematic review and comparative frequentist and Bayesian meta-analysis of randomized controlled trials
Source: Ann Intensive Care. 2024 Sep 28;14:150. doi: 10.1186/s13613-024-01376-1 (PMC11438751; doi:10.1186/s13613-024-01376-1)
Supplement: Supplementary file 1 — Additional file 1. [file 13613_2024_1376_MOESM1_ESM.docx]

## Supplentary Table 1: Summary of the quality of evidence according to the Grading of Recommendation Assessment, Development, and Evaluation (GRADE)

| **Patient or population:** Adult patients with acute myocardial infarction  **Settings:** In-hospital care  **Intervention:** Liberal blood transfusion  **Comparison:** Restrictive blood transfusion | | | | | | |
| --- | --- | --- | --- | --- | --- | --- |
| **Outcomes**  **No of participants (studies)** | **Risk Ratio** | **Anticipated absolute effects** | | | **Certainty of the evidence (GRADE)** | **Comments** |
|  |  | Restrictive | Liberal | Absolute differ-ence |  |  |
| Mortality  (4 studies,  n=4324) | 1.13  [0.67, 1.91] | 9.3% | 8.0% | - 1.3% | ⊕⊕⊝⊝  **Low**^a,c^ | May or may not decrease in-hospital mortality |
| MI or death  (3 studies,  n=3658) | 1.18  [1.01; 1.37] | 16.9% | 14.4% | 2.5% | ⊕⊝⊝⊝  **Very low**^a,c,d^ | May decrease MI or Death |
| MI  (4 studies,  n=4324) | 1.16  [0.94; 1.45] | 7.6% | 6.6% | 1.0% | ⊕⊕⊝⊝  **Low**^a,c^ | May or may not decrease MI |
| Cardiac death  (2 studies,  n=4170) | 1.05  [0.36; 3.04] | 5.2% | 3.7% | 1.55% | ⊕⊝⊝⊝  **Very low**^a,b,e^ | May or may not decrease cardiac death |
| Revascularization  (3 studies,  n=4279) | 1.06  [0.72; 1.57] | 2.3% | 2.2% | 0.12% | ⊕⊝⊝⊝  **Very low**^a,c,e,f^ | May or may not decrease occurence of revascularization |
| Stroke  (3 studies,  n=4279) | 1.11  [0.67; 1.82] | 1.4% | 1.3% | 0.13% | ⊕⊝⊝⊝  **Very low**^a,b,f^ | May or may not decrease occurence of stroke |

1. Downgraded due to **imprecision** with a small number of studies.
2. Downgraded due to **inconsistency** because we detected heterogeneity / inconsistency results
3. Downgraded due **risk of bias** because one or two of study is jugged at high risk of bias
4. Downgraded due to **imprecision**, with the effect being primarily driven by a single study.
5. Downgraded due to **publication bias** because asymmetric funnel plot
6. Downgraded due to **imprecision** because low incidence outcome
