# Supplementary material for: Liberal versus restrictive transfusion strategies in acute myocardial infarction: a systematic review and comparative frequentist and Bayesian meta-analysis of randomized controlled trials
Source: Ann Intensive Care. 2024 Sep 28;14:150. doi: 10.1186/s13613-024-01376-1 (PMC11438751; doi:10.1186/s13613-024-01376-1)
Supplement: Supplementary file 5 — Additional file 5. [file 13613_2024_1376_MOESM5_ESM.docx]

| Study | Carson *et al .* 2023 |
| --- | --- |
| Participants | **Total number of randomized participants :** 3504  **Inclusion criteria :** Adults patients with myocardial infarction with anemia < 10g/dl within 24 hours before randomization. Patients with type 1, 2, 4b, or 4c myocardial infarction were eligible for enrollment  **Exclusion criteria :** Uncontrolled bleeding, palliative treatment, scheduled for cardiac surgery, patient declined to receive blood transfusion  **Definition of myocardial infarction:** Third Universal Definition of Myocardial Infarction**^a^**  **Baseline characteristics restrictive group :**   - Age, mean (SD) : 72.2 (11.5) years - Female sex: 44.3 percent - Ethnic group, percent : white (70.3), black (12.4) - NSTEMI : 81.8 percent - Type 1 : 41.7 percent - Type 2 : 55.3 percent - Revascularisation before randomization : 29.1 percent - In hospital heart failure before randomization : 21.6 percent - Mechanical ventilation before randomization : 14.3 percent   **Baseline characteristics liberal group :**   - Age, mean (SD) : 72.1 (11.6) years - Female sex: 46.7 percent - Ethnic group, percent : white (70.9), black (12.7) - NSTEMI : 80.8 percent - Type 1 : 41.6 percent - Type2 : 56.3 percent - Revascularisation before randomization : 28.1 percent - In hospital heart failure before randomization : 23 percent - Mechanical ventilation before randomization : 13.2 percent   **Country :** United States, Canada, France, Brazil, New Zealand, and Australia  **Number of sites :** 144 |
| Interventions | **Restrictive group** :   - n = 1749 - Détails : Blood transfusion was permitted under 8 g/dl and strongly recommanded under 7 g.dl or when anginal symptoms were not controlled with medications   **Liberal group** :   - n = 1755 - Détails : One unit of packed red cells was administered after randomization and red cells were transfused to maintain the hemoglobin level at or above 10 g per deciliter |
| Outcomes | Mortality, MI, myocardial infarction, stroke, revascularization at 30 days |
| Notes | **Study dates** : from April 2017 through April 2023 |

a : Thygesen K, Alpert JS, Jaffe AS, et al. Third universal definition of myocardial infarction. Circulation 2012;126:2020-35

| Study | Ducrocq *et al .* 2021 |
| --- | --- |
| Participants | **Total number of randomized participants :** 668  **666 patients completed follow up**  **Inclusion criteria :** Adults patients with myocardial infarction within 48 hours before admission and a hemoglobin level between 7 and 10 g/dL. Enrollment could be considered at any time during the index admission for myocardial infarction  **Exclusion criteria :** Shock, myocardial infarction occurring after percutaneous coronary intervention or coronary artery bypass graft**,** blood transfusion in the past 30 days, and malignant hematologic disease  **Definition of myocardial infarction :** Third Universal Definition of Myocardial Infarction**^a^**  **Baseline characteristics restrictive group :**   - Age, median (IQR) : 78 (69-85) years - Female sex: 41.2 percent - Ethnic group, percent : white (88.7), black (2.1) African/Caribbean 7 (2.1) Indian 2 (0.6) Other Asian 0 - NSTEMI : 68.4 percent - STEMI : 31.6 percent - Type 1 : NI - Type2 : NI - Revascularisation before randomization : 58.8 percent - In hospital heart failure before randomization : NI - Mechanical ventilation before randomization : NI   **Baseline characteristics liberal group :**   - Age, mean (SD) : 76 (69-84) years - Female sex: 43.2 percent - Ethnic group, percent : white (82.6), black (2.8) African/Caribbean 9 (2.8), Indian 5 (1.6) Other Asian 6 (1.9) - NSTEMI : 71.3 percent - STEMI : 28.7 percent - Type 1 : NI - Type2 : NI - Revascularisation before randomization : 59.3 percent - In hospital heart failure before randomization : NI - Mechanical ventilation before randomization : NI   **Country :** France, Spain  **Number of sites :** 35 |
| Interventions | **Restrictive group** :   - 342 Included in as-randomized analyses - 327 Included in as-treated analyses - Détails : No transfusion unless hemoglobin level decreased to less than or equal to 8 g/dL, with a target range for post-transfusion hemoglobin of 8 to 10 g/dL.   **Liberal group** :   - 324 Included in as-randomized analyses - 322 Included in as-treated analyses - Détails : Transfusion to be performed after randomization on all patients with a hemoglobin level less than or equal to 10 g/dL, with a target post-transfusion hemoglobin level of at least 11 g/dL |
| Outcomes | Mortality, MI, myocardial infarction, cardiac death, stroke, revascularization at 30 days |
| Notes | **Study dates** : From March 2016 through September 2019 |

a : Thygesen K, Alpert JS, Jaffe AS, et al. Third universal definition of myocardial infarction. Circulation 2012;126:2020-35

| Study | Carson *et al .* 2013 |
| --- | --- |
| Participants | **Total number of randomized participants :** 110  **Inclusion criteria :** Adult patients with myocardial infarction or unstable angina, or stable coronary artery disease undergoing cardiac catheterization. Hemoglobin levels below 10g/dL at randomization.  **Exclusion criteria :** Active bleeding. Hemodynamic instability at any point during the index admission. Other health issues that could interfere with symptom reporting or adherence to treatment protocols  **Definition of myocardial infarction :**   - MI : The Joint European Society of Cardiology/American College of Cardiology Committee definitions^a^ - STEMI : Symptoms of rest ischemia ≥10 min; ST-elevation ≥1 mm or new left bundle-branch block; elevated cardiac biomarkers. - NSTEMI : Rest ischemia symptoms ≥10 min; troponin or creatine kinase MB above normal. - Unstable angina : Rest ischemia symptoms ≥10 min; ST-depression or transient elevation; possible coronary disease history. - Stable coronary disease : Presence of ≥70% coronary artery obstruction or undergoing cardiac intervention during admission.   **Baseline characteristics restrictive group :**   - Age, mean (SD) : 74.3 (11.1) years - Female sex: 50.9 percent - Ethnic group, percent : white (74.6), black (21.8) - NSTEMI : 47.3 percent - STEMI: 29.1 percent - Type 1 : NI - Type2 : NI - Unstable angina : 14.6 percent - Stable coronary artery disease : 9.1 percent - Revascularisation before randomization : 47.3 percent - In hospital heart failure before randomization : 23.6 percent - Mechanical ventilation before randomization : 0 percent   **Baseline characteristics liberal group :**   - Age, mean (SD) : 67.3 (13.6) years - Female sex: 49.1 percent - Ethnic group, percent : white (70.9), black (18.2) - NSTEMI : 38.2 percent - STEMI : 30.9 percent - Type 1 : NI - Type2 : NI - Unstable angina : 14.6 percent - Stable coronary artery disease : 16.4 percent - Revascularisation before randomization : 63.6 percent - In hospital heart failure before randomization : 21.8 percent - Mechanical ventilation before randomization : 0 percent   **Country :** US  **Number of sites :** 8 |
| Interventions | **Restrictive group** :   - n = 54 - Détails : Transfusion allowed for anemia-related symptoms. Permitted but not required if hemoglobin <8 g/dL. No mandatory lower threshold. Transfusion one unit at a time to relieve symptoms or raise hemoglobin >8 g/dL.   **Liberal group** :   - n = 55 - Détails : One unit of red blood cells post-randomization. Objectif to maintain hemoglobin ≥10 g/dL. Transfusion if <10 g/dL during hospitalization up to 30 days. |
| Outcomes | Mortality, MI, myocardial infarction, stroke, revascularization at 30 days |
| Notes | **Study dates** : From March 2010 through May 2012 |

a : Thygesen K, Alpert JS, White HD, et al. Universal definition of myocardial infarction. Circulation 2007;116:2634-53.

| Study | Cooper *et al .* 2011 |
| --- | --- |
| Participants | **Total number of randomized participants :** 45  **Inclusion criteria :** Adult patients myocardial infarction and hematocrit ≤30% within 72 hours of symptom onset  **Exclusion criteria :** Non-coronary cause of the clinical syndrome, active bleeding defined by overt blood loss with a hematocrit decrease of ≥5% in the preceding 12 hours, inability or unwillingness to receive RBC transfusion, RBC transfusion within 7 days prior to enrollment, previous severe transfusion reaction, imminent death, decision to provide limited or comfort care, age <21 years, pregnancy, participation in another clinical trial involving RBC transfusion, and previous participation in the study.  **Definition of myocardial infarction :**   - MI : Acute myocardial infarction (AMI) is defined as ischemic-type chest discomfort persisting for 30 minutes or more, accompanied by elevated levels of creatine kinase-MB (CK-MB) or cardiac troponin, surpassing the locally established upper normal limit   **Baseline characteristics restrictive group :**   - Age, mean (SD) : 70.3 (14.3) years - Men sex: 54 percent - Ethnic group, percent : white (61) - NSTEMI : 54 percent - STEMI: 46 percent - Type 1 : NI - Type2 : NI - Unstable angina : NI - Stable coronary artery disease : NI - Revascularisation before randomization : 54 percent - In hospital heart failure before randomization : NI - Mechanical ventilation before randomization : 13 percent   **Baseline characteristics liberal group :**   - Age, mean (SD) : 76.4 (13.5) years - Female sex: 48 percent - Ethnic group, percent : white (76) - NSTEMI : 67 percent - STEMI : 33 percent - Type 1 : NI - Type2 : NI - Unstable angina : NI - Stable coronary artery disease : NI - Revascularisation before randomization : 57 percent - In hospital heart failure before randomization : NI - Mechanical ventilation before randomization : 24 percent   **Country :** US  **Number of sites :** 3 |
| Interventions | **Restrictive group** :   - n = 24 - Détails : Transfusion was initiated when hematocrit fell below 24%, aiming to maintain levels between 24% and 27%   **Liberal group** :   - n = 21 - Détails : Transfusion was initiated when hematocrit fell below 30%, aiming to maintain levels between 30% and 33%. |
| Outcomes | Mortality, MI, myocardial infarction |
| Notes | **Study dates** : From May 2003 through October 2009 |

| Study | Carson et al. 2023 | Ducrocq et al. 2021 | Carson et al. 2013 | Cooper et al. 2011 |
| --- | --- | --- | --- | --- |
| Participants | 3504 | 666 | 110 | 45 |
| Inclusion Criteria | Myocardial infarction with anemia < 10g/dl | Myocardial infarction, Hb between 7-10 g/dL | Myocardial infarction, unstable angina, stable coronary disease, Hb < 10g/dL | Myocardial infarction, hematocrit ≤30% |
| Exclusion Criteria | Uncontrolled bleeding, palliative treatment, scheduled cardiac surgery, decline of blood transfusion | Shock myocardial infarction post-PCI or CABG, blood transfusion in last 30 days, malignant hematologic disease | Active bleeding, hemodynamic instability, health issues interfering with symptom reporting or treatment adherence | Non-coronary cause, active bleeding (≥5% hematocrit drop in 12 hours), unwillingness/inability to receive RBC transfusion, recent RBC transfusion (within 7 days), severe transfusion reaction history, imminent death, limited/comfort care decision, age <21 |
| Baseline Characteristics (Restrictive Group) | Age: 72.2 years  Female: 44.3%  Ethnicity: 70.3% white NSTEMI: 81.8%  Type 1: 41.7%  Type 2: 55.3%  Revascularization: 29.1%  Heart failure: 21.6%  Dialysis: 11.8%  Mechanical ventilation: 13.7% | Age: 78 years  Female: 41.2%  Ethnicity: 88.7% white NSTEMI: 68.4%  Revascularization: 58.8%  Dialysis: 8%  Mechanical ventilation: NA | Age: 74.3 years  Female: 50.9%  Ethnicity: 74.6% white NSTEMI: 47.3%  Revascularization: 47.3% Heart failure: 23.6%  Dialysis: NA  Mechanical ventilation: NA | Age: 70.3 years  Female:46 %  Ethnicity: 61 % white  NSTEMI: 54 %  Revascularization: 54 %  Heart failure: NA  Dialysis: NA  Mechanical ventilation: 13% |
| Baseline Characteristics (Liberal Group) | Age: 72.1 years  Female: 46.7%  Ethnicity: 70.9% white NSTEMI: 80.8%  Type 1: 41.6%  Type 2: 56.3%, Revascularization: 28.1% Heart failure: 23% | Age: 76 years  Female: 43.2%  Ethnicity: 82.6% white  NSTEMI: 71.3%,  Revascularization: 59.3% | Age: 67.3 years  Female: 49.1%  Ethnicity: 70.9% white  NSTEMI: 38.2%  Revascularization: 63.6%Heart failure: 21.8% | Age: 76.4 years  Female:52 %  Ethnicity: 76 % white  NSTEMI: 67 %  Revascularization: 57 %  Heart failure: NA  Dialysis: NA  Mechanical ventilation: 24% |
| Hemoglobin levels | Baseline:  8.6 g/dl for restrictive  8.6 g/dl liberal  Days 3:  8.9 g/dl for restrictive  10.5 g/dl liberal  Discharge: NA | Baseline:  9 g/dl for restrictive  9.1 g/dl liberal  Days 3: NA  Discharge:  9.7 g/dl for restrictive  11,1 g/dl for liberal | Baseline:  9.03 g/dl for restrictive  10.3 g/dl liberal  Days 3:  9.12 g/dl for restrictive  10.64 g/dl liberal  Discharge: NA | Baseline:  27.5 % for restrictive  26.9% liberal  Days 3:  ~ 27-28 % for restrictive  ~ 32-33 % for liberal  Discharge:  ~ 27-28 % for restrictive  ~ 28-30 % for liberal |
| ICU at randomization | 47.9% | NA | NA | NA |
| Countries and Sites | US, Canada, France, Brazil, New Zealand, Australia (144 sites) | France, Spain (35 sites) | US (8 sites) | US (3 sites) |
| Group Details (Restrictive) | Blood transfusion permitted under 8 g/dl | No transfusion unless Hb ≤ 8 g/dL, target post-transfusion Hb 8-10 g/dL | One unit of RBCs post-randomization, transfusion if Hb <10 g/dL | Transfusion was initiated when hematocrit fell below 24%, aiming to maintain levels between 24% and 27% |
| Group Details (Liberal) | One unit of RBCs post-randomization, maintain Hb ≥ 10 g/dL | Transfusion for Hb ≤ 10 g/dL, target post-transfusion Hb ≥ 11 g/dL | Transfusion allowed for anemia-related symptoms, no mandatory lower threshold | Transfusion was initiated when hematocrit fell below 30%, aiming to maintain levels between 30% and 33%. |
| Overall risk of bias  (Rob-2) | Low risk of bias | Low risk of bias | High risk of bias | High risk of bias |
| Study Dates | April 2017 - April 2023 | March 2016 - September 2019 | March 2010 - May 2012 | May 2003 - October 2009 |
