# Supplementary figures and images for: Liberal versus restrictive transfusion strategies in acute myocardial infarction: a systematic review and comparative frequentist and Bayesian meta-analysis of randomized controlled trials
Source: Ann Intensive Care. 2024 Sep 28;14:150. doi: 10.1186/s13613-024-01376-1 (PMC11438751; doi:10.1186/s13613-024-01376-1)

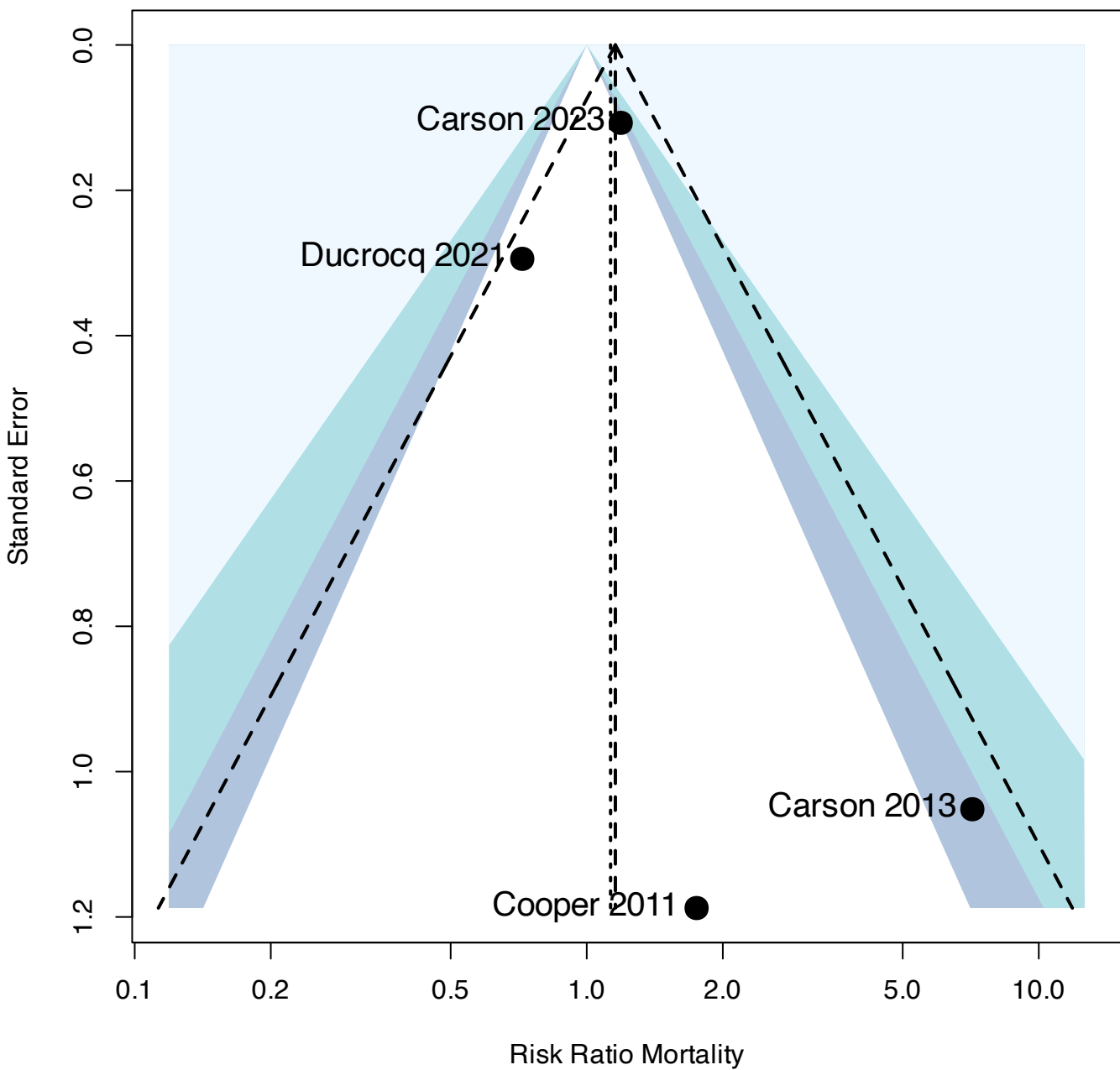

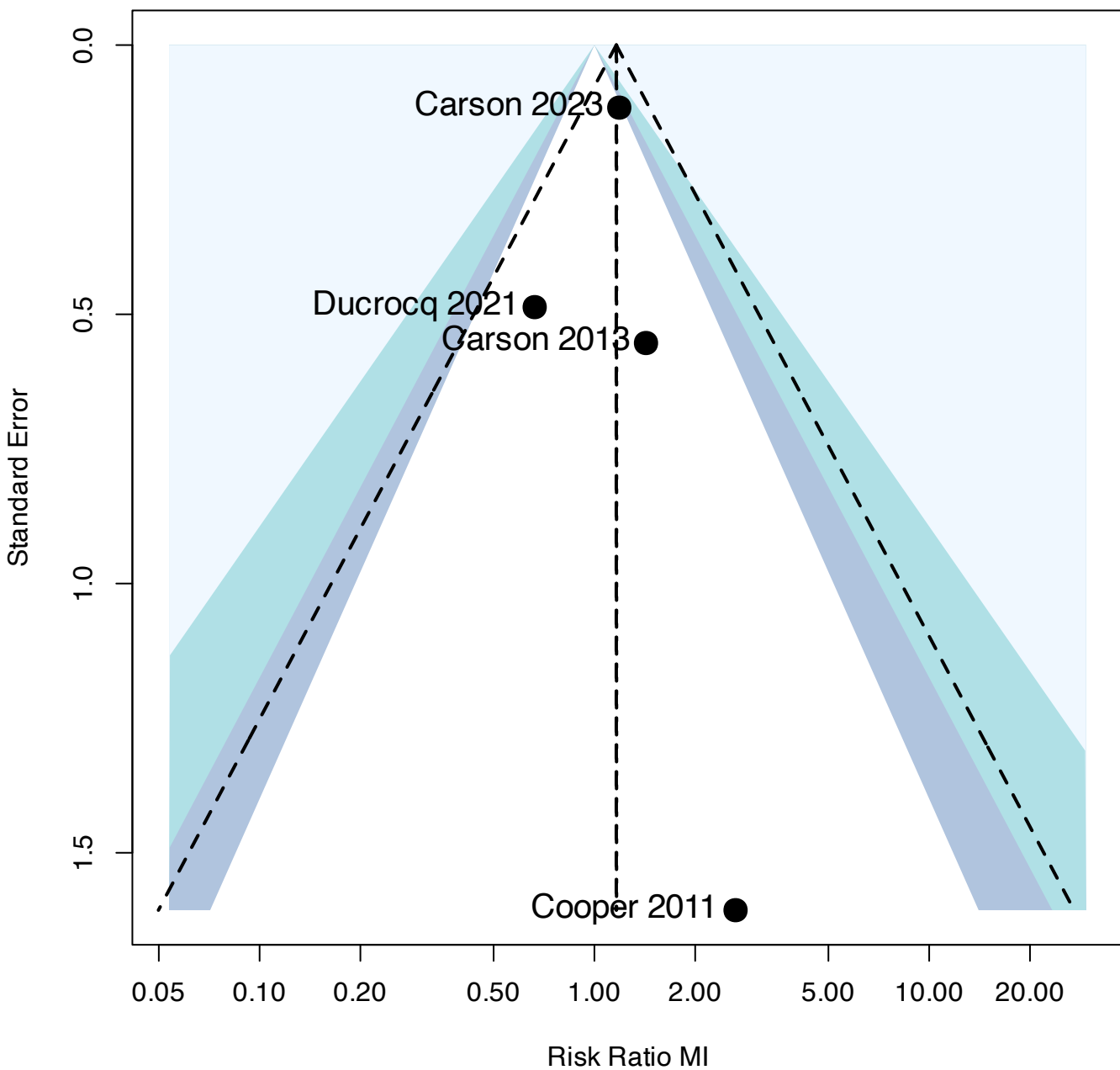

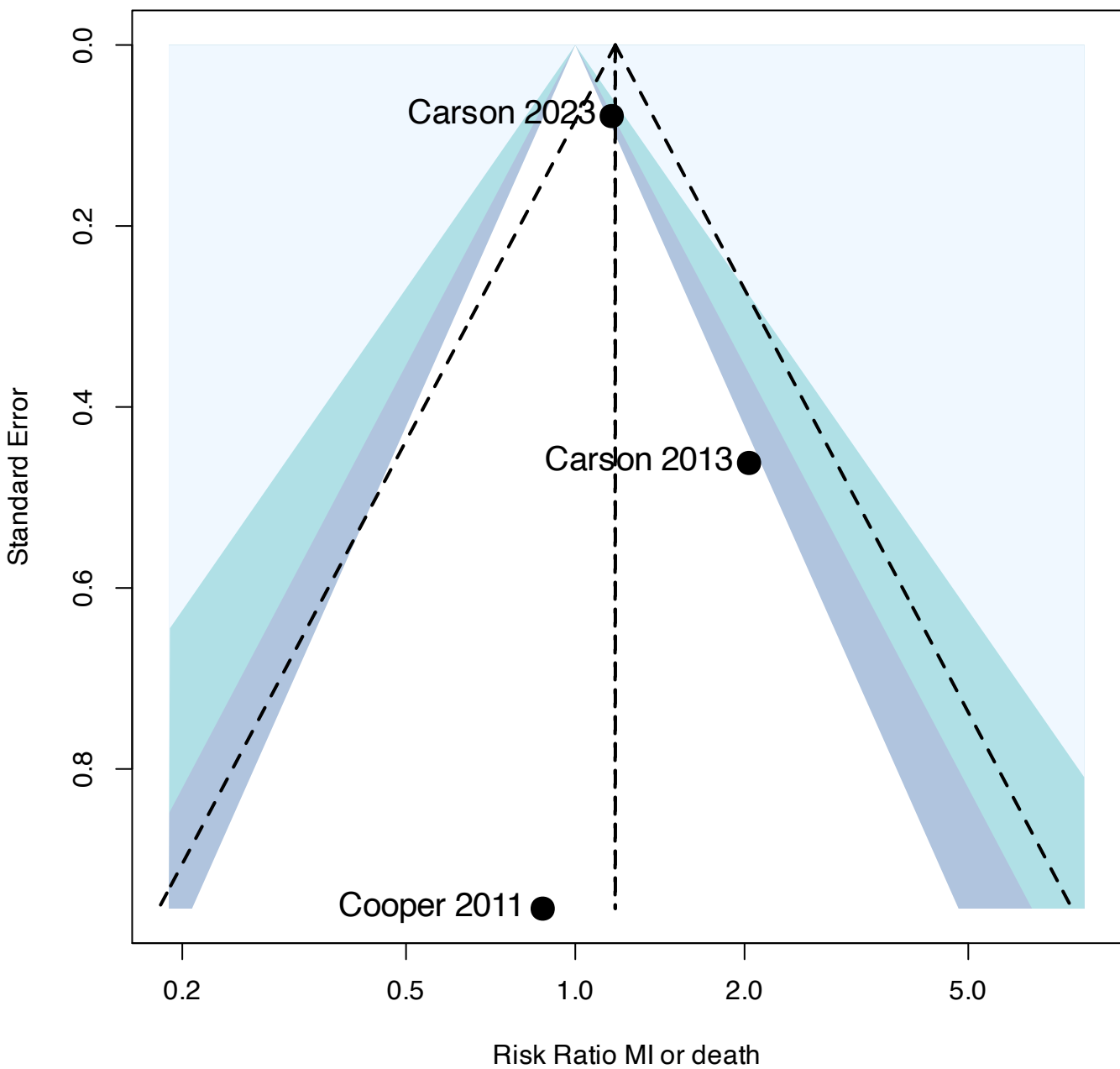

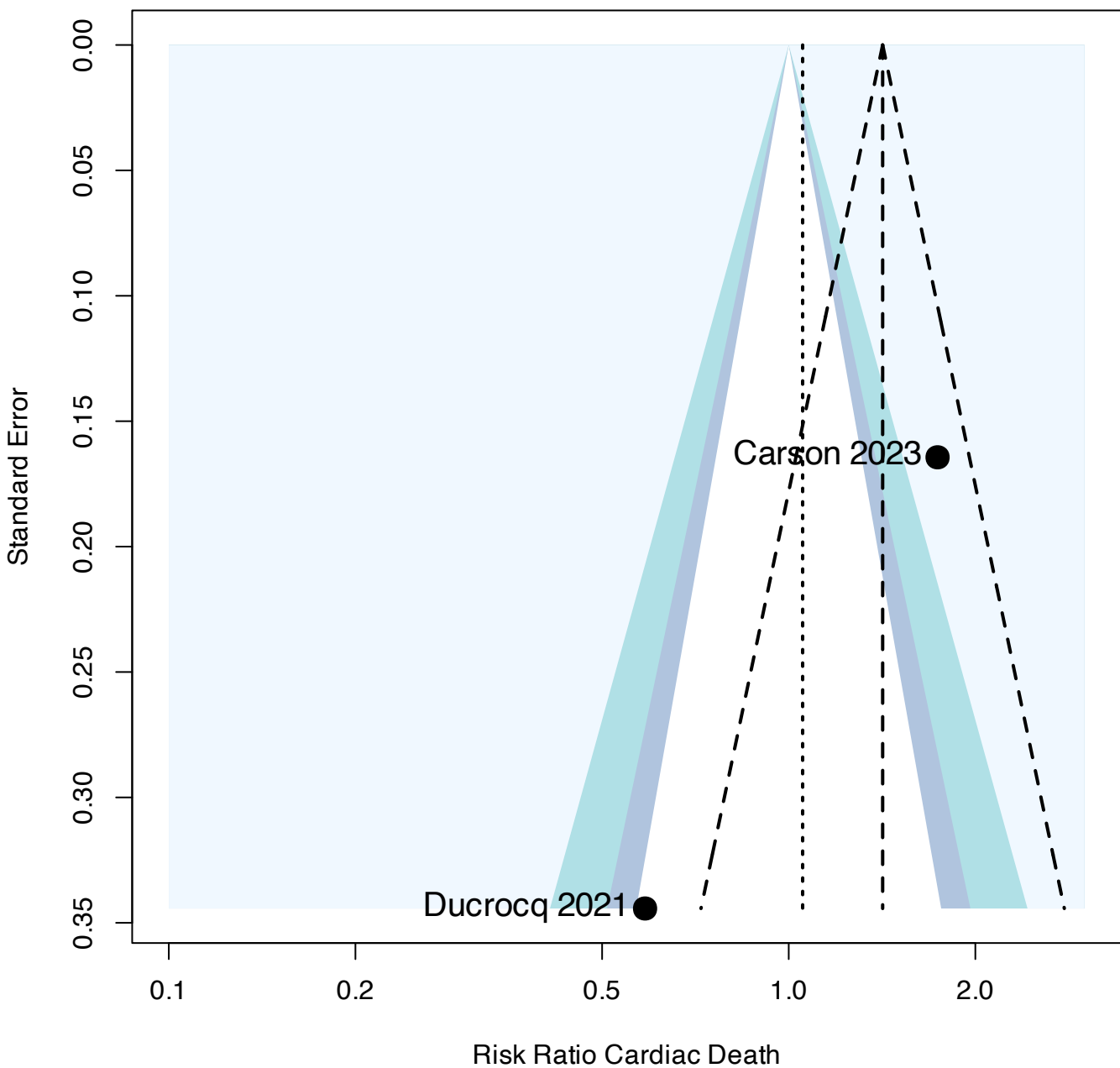

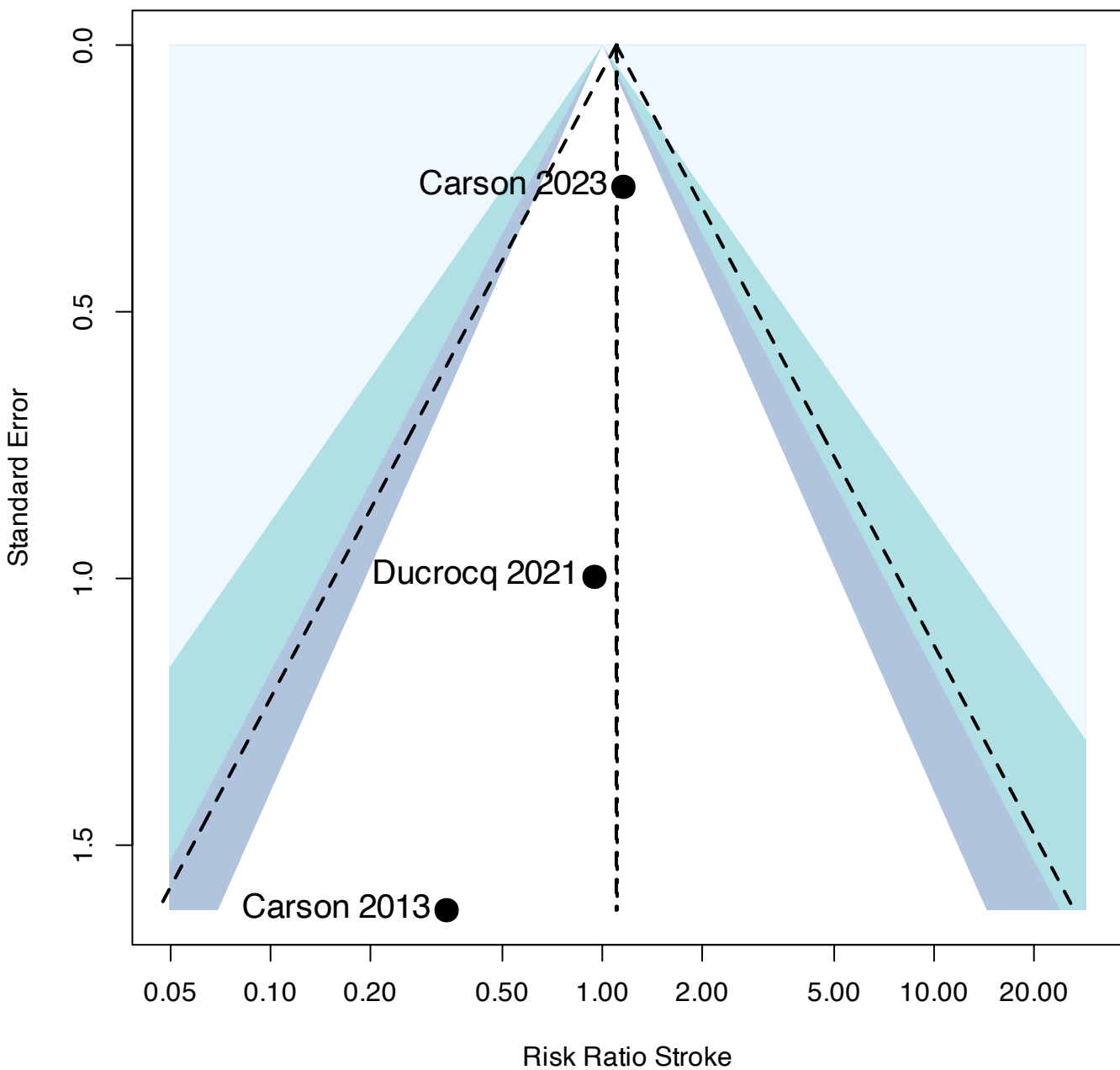

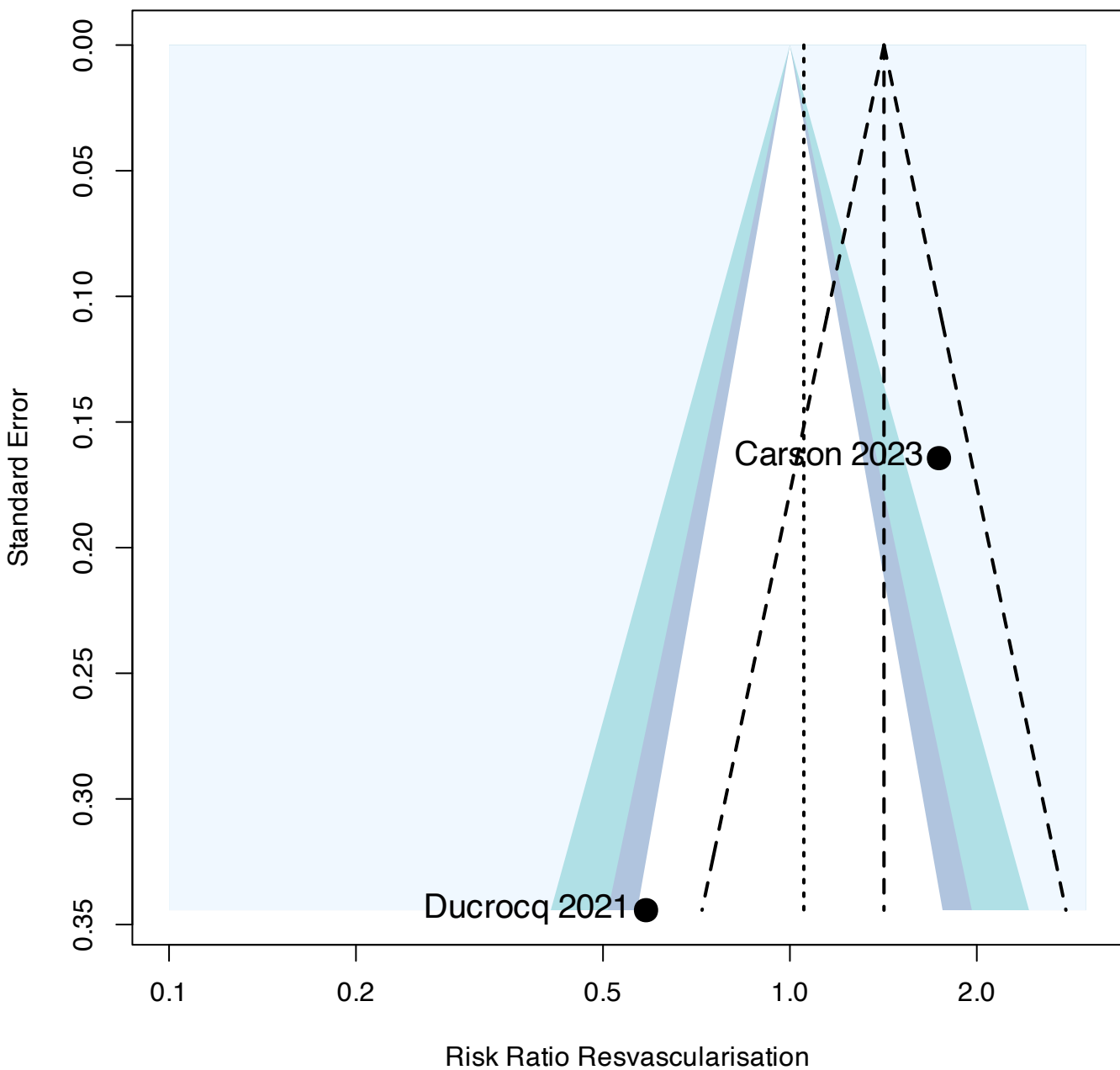

Supplement: Supplementary file 8 — Additional file 8. Figure S1. Funnel Plot Analysis for Heterogeneity and Publication Bias Assessment. This funnel plot was employed to visually assess heterogeneity and potential publication bias in our meta-analysis. [file 13613_2024_1376_MOESM8_ESM.pdf]
