# Supplementary material for: Liberal versus restrictive transfusion strategies in acute myocardial infarction: a systematic review and comparative frequentist and Bayesian meta-analysis of randomized controlled trials
Source: Ann Intensive Care. 2024 Sep 28;14:150. doi: 10.1186/s13613-024-01376-1 (PMC11438751; doi:10.1186/s13613-024-01376-1)
Supplement: Supplementary file 9 — Additional file 9. Figure S2. Rob2 Risk-of-Bias Visualization. This graphic displays the risk-of-bias for each study and each outcome in our meta-analysis as per Rob2 criteria (revised Cochrane risk-of-bias tool for randomized trials). It categorizes bias risk levels across key domains for each study, allowing for a quick comparative assessment of their methodological quality. [file 13613_2024_1376_MOESM9_ESM.pdf]

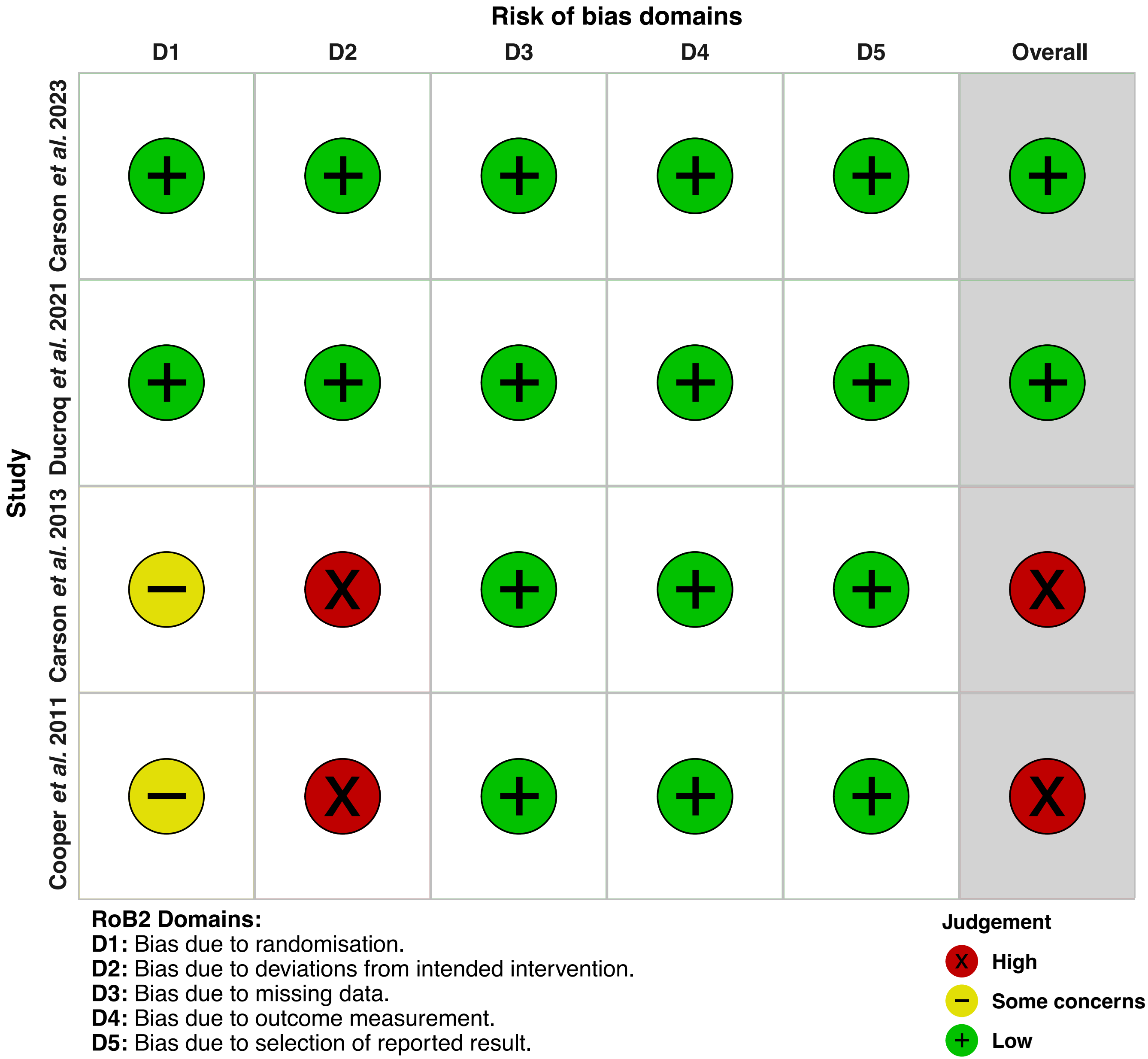
Risk of bias domains
D1
D2
D3
D4
D5
Overall
Study
Carson et al. 2023
Ducroq et al. 2021
Carson et al. 2013
Cooper et al. 2011
RoB2 Domains:
D1: Bias due to randomisation.
D2: Bias due to deviations from intended intervention.
D3: Bias due to missing data.
D4: Bias due to outcome measurement.
D5: Bias due to selection of reported result.
Judgement
X High
− Some concerns
+ Low

Mortality

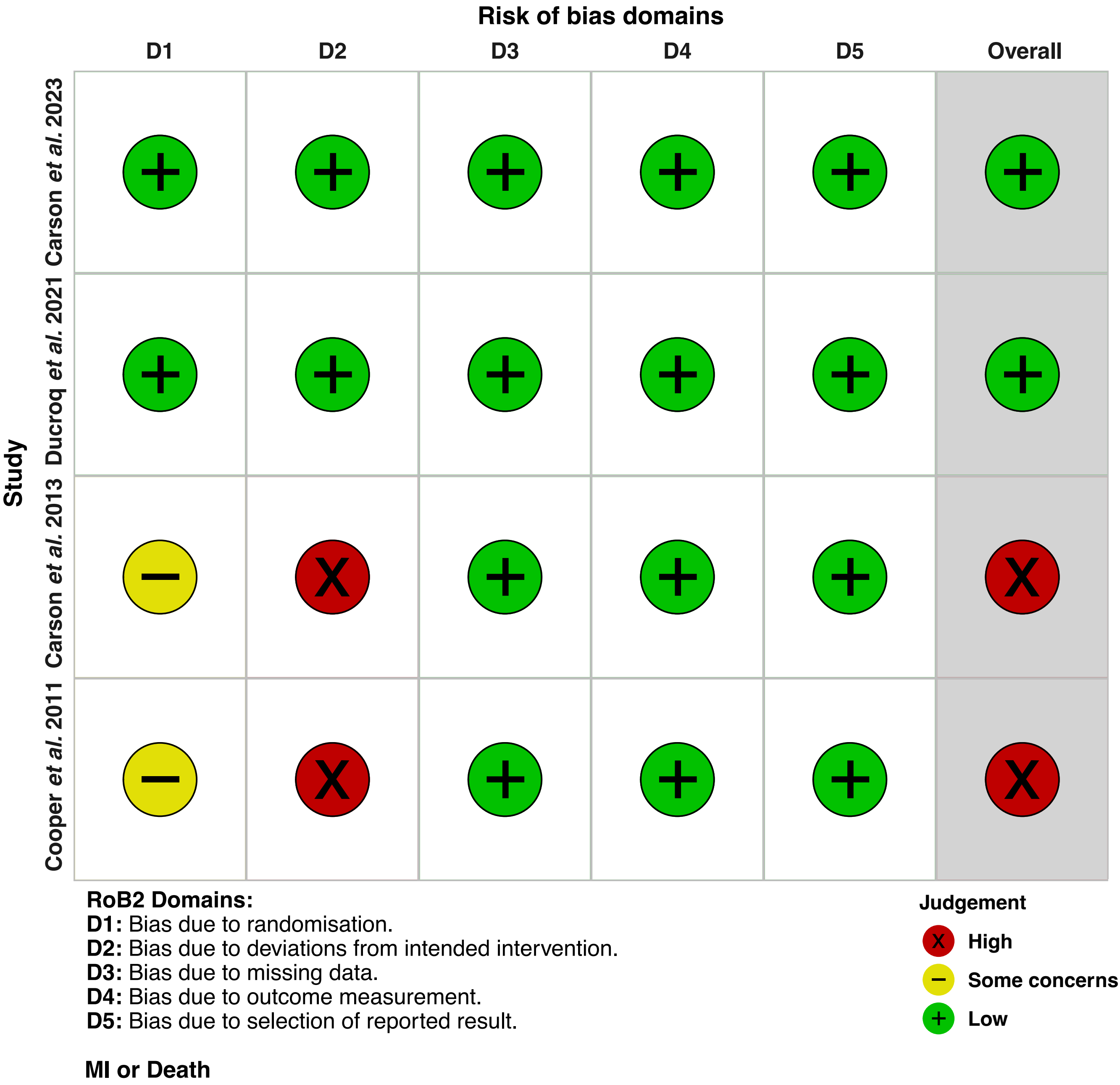
Risk of bias domains
D1
D2
D3
D4
D5
Overall
Study
Carson et al. 2023
Ducroq et al. 2021
Carson et al. 2013
Cooper et al. 2011
RoB2 Domains:
D1: Bias due to randomisation.
D2: Bias due to deviations from intended intervention.
D3: Bias due to missing data.
D4: Bias due to outcome measurement.
D5: Bias due to selection of reported result.
MI or Death
Judgement
High
Some concerns
Low

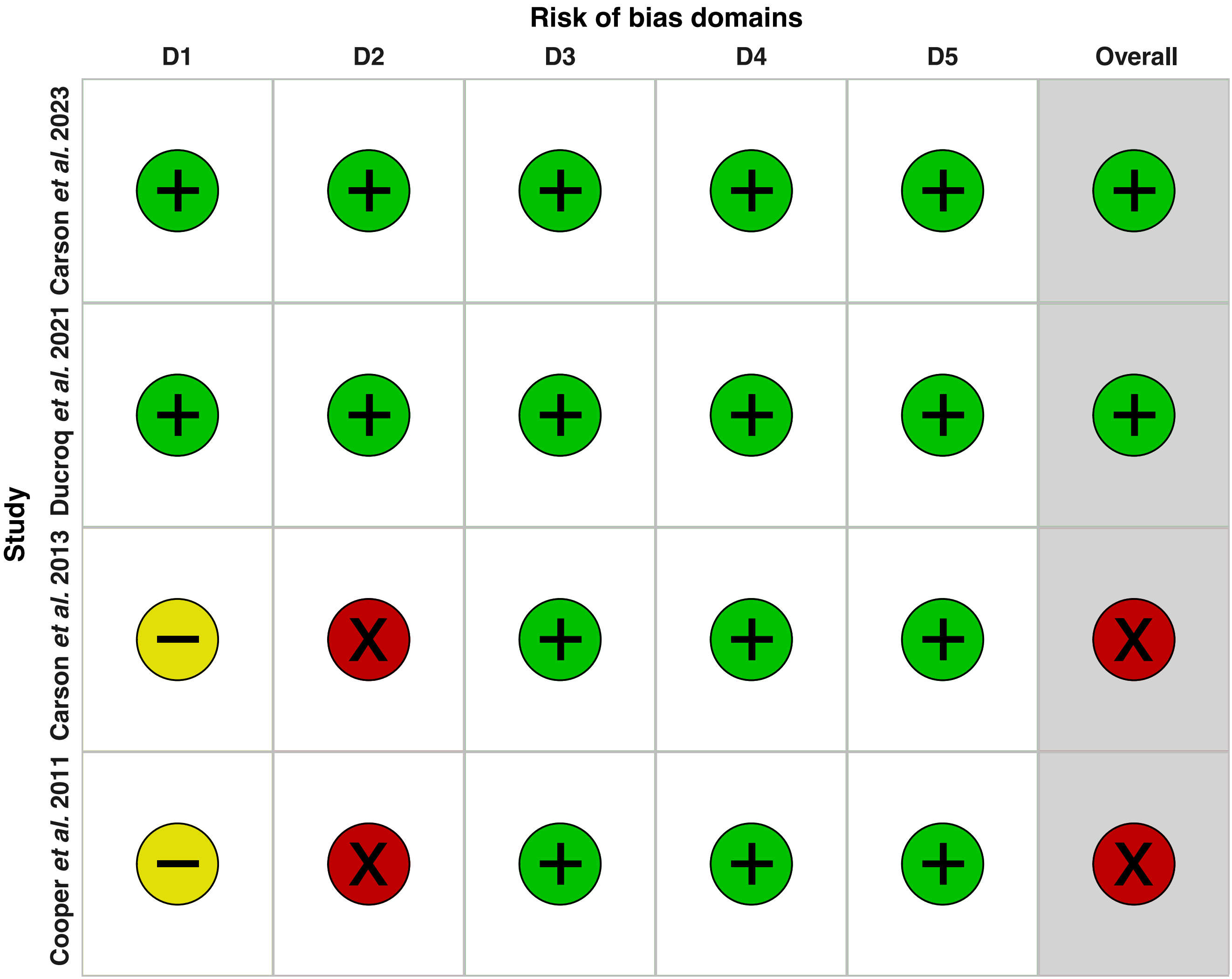

RoB2 Domains:
D1: Bias due to randomisation.
D2: Bias due to deviations from intended intervention.
D3: Bias due to missing data.
D4: Bias due to outcome measurement.
D5: Bias due to selection of reported result.

Judgement
X High
− Some concerns
+ Low

Myocardial infarction

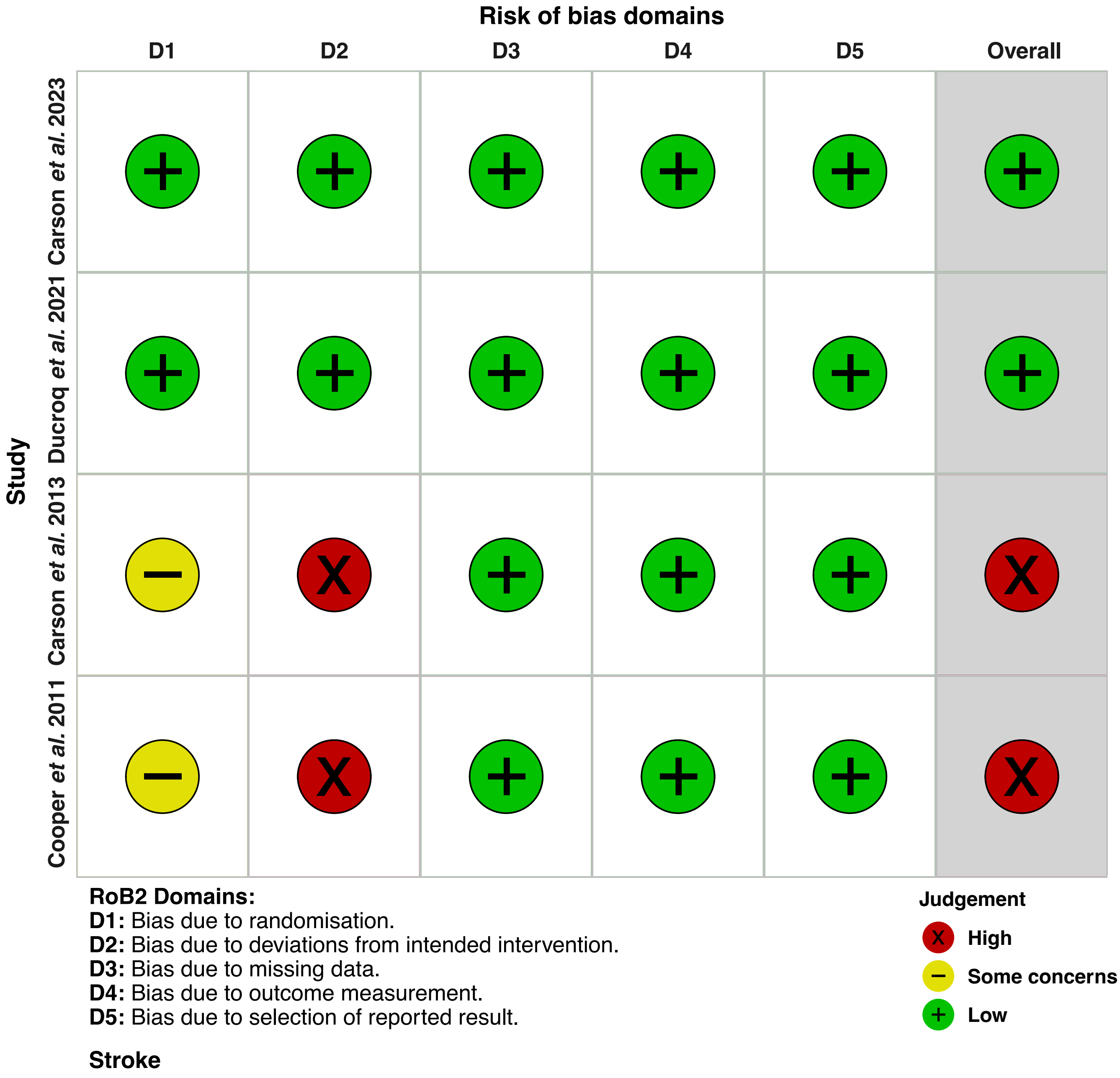

Risk of bias domains
D1
D2
D3
D4
D5
Overall
Study
Carson et al. 2023
Ducroq et al. 2021
Carson et al. 2013
Cooper et al. 2011
RoB2 Domains:
D1: Bias due to randomisation.
D2: Bias due to deviations from intended intervention.
D3: Bias due to missing data.
D4: Bias due to outcome measurement.
D5: Bias due to selection of reported result.
Stroke
Judgement
X High
− Some concerns
+ Low

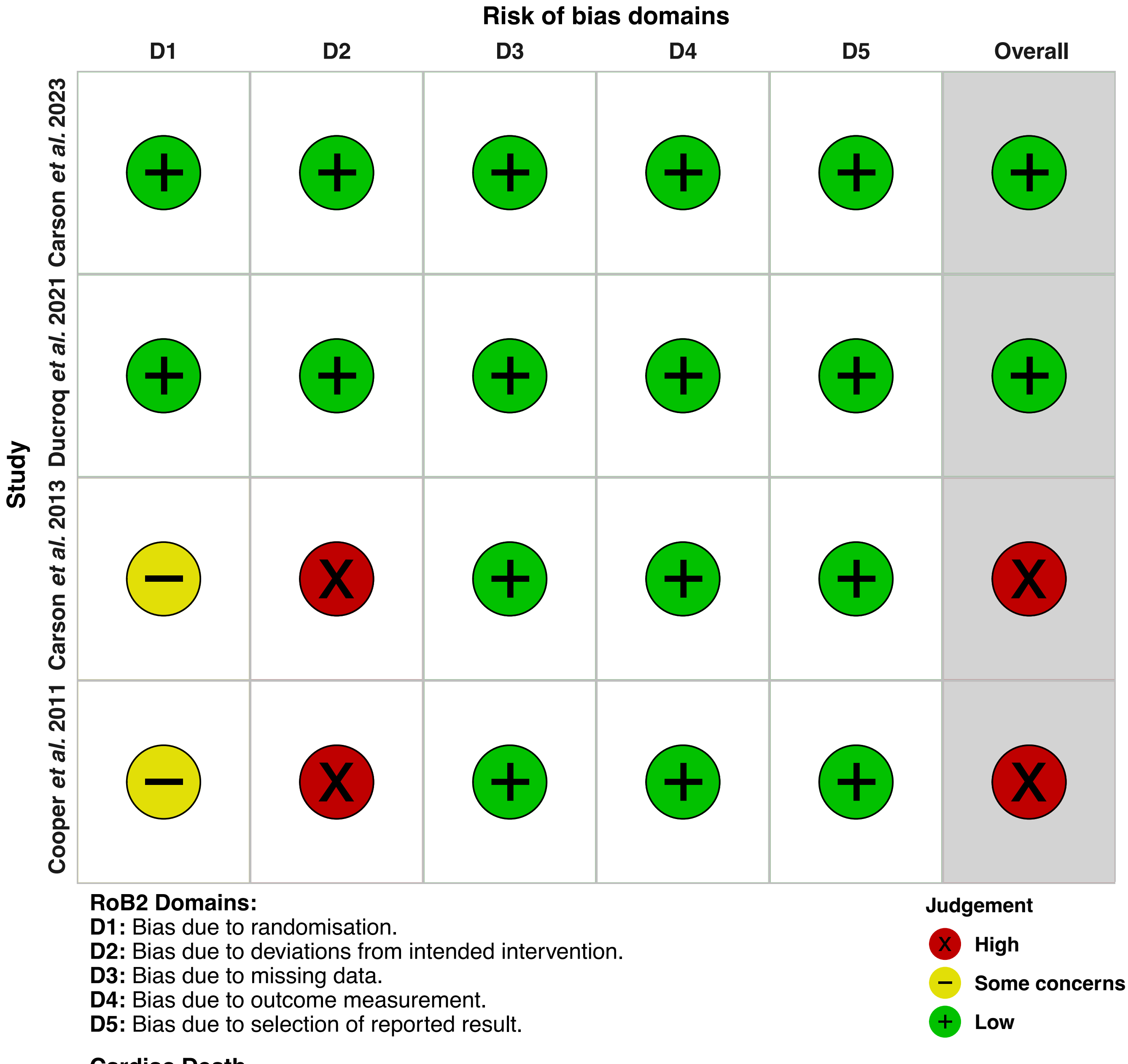

RoB2 Domains:
D1: Bias due to randomisation.
D2: Bias due to deviations from intended intervention.
D3: Bias due to missing data.
D4: Bias due to outcome measurement.
D5: Bias due to selection of reported result.

Cardiac Death

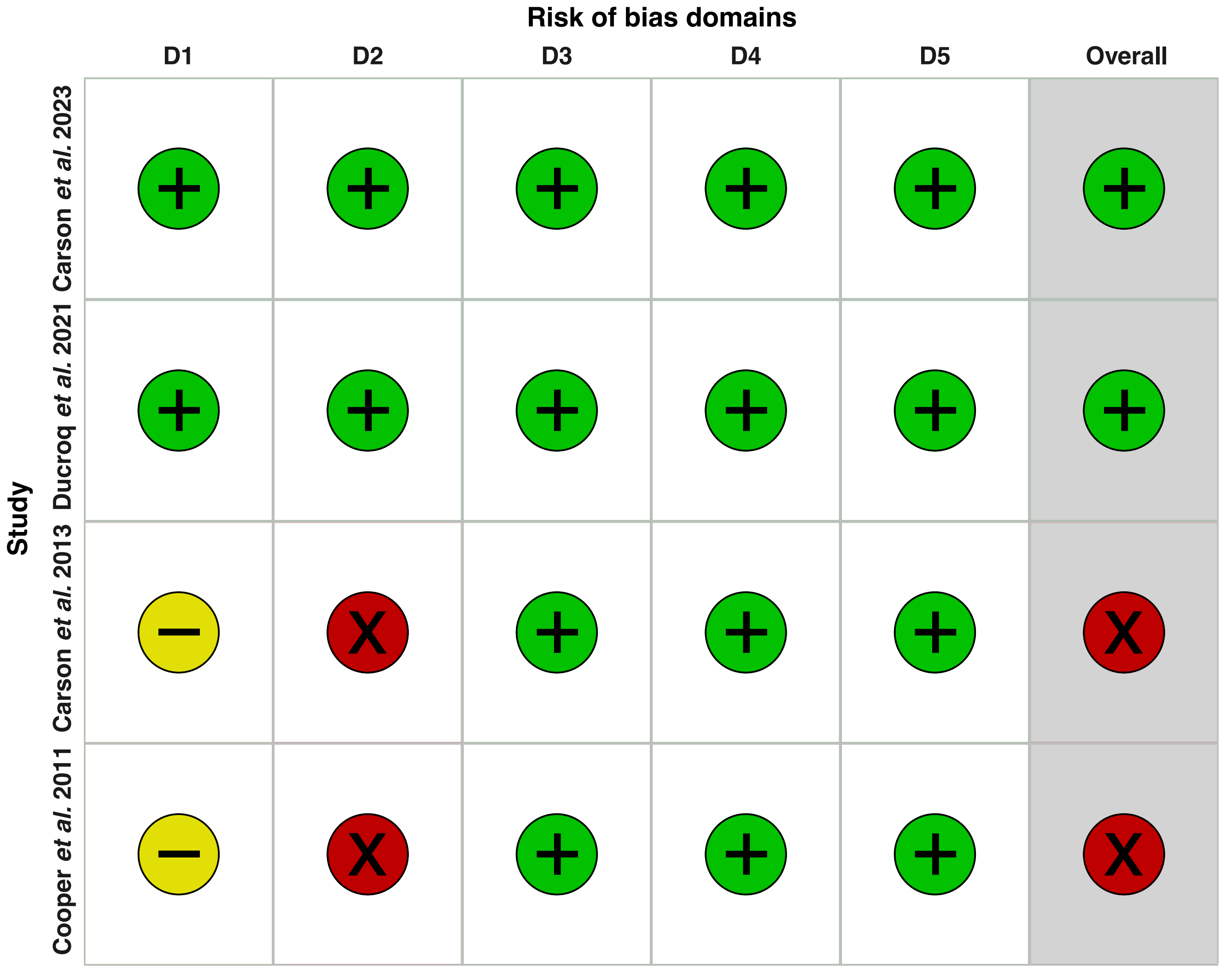

RoB2 Domains:
D1: Bias due to randomisation.
D2: Bias due to deviations from intended intervention.
D3: Bias due to missing data.
D4: Bias due to outcome measurement.
D5: Bias due to selection of reported result.

Judgement
X High
− Some concerns
+ Low

Revascularization
